# Hospital-at-home for acute complicated diverticulitis: a single-arm feasibility study

**DOI:** 10.1007/s00464-025-12048-x

**Published:** 2025-08-29

**Authors:** Neyla Boukhili, Tiffany Paradis, Sarah Faris-Sabboobeh, Marie Demian, Nancy Morin, Carol-Ann Vasilevsky, Gabriela Ghitulescu, Julio Faria, Allison Pang, Marylise Boutros

**Affiliations:** 1 Department of Surgery, Division of General Surgery, McGill University, Montreal, QC Canada; 2 Division of Colon and Rectal Surgery, Jewish General Hospital, Montreal, QC Canada; 3 Department of Colorectal Surgery, Cleveland Clinic Florida, Weston Hospital, Ellen Leifer Shulman and Steven Shulman Digestive Disease Center, 2950 Cleveland Clinic Boulevard, Weston, FL 33331 USA

**Keywords:** Non-operative management, Hospital-at-home program, Acute complicated diverticulitis, Feasibility study

## Abstract

**Introduction:**

Non-operative management (NOM) of acute complicated diverticulitis (ACD) has become the preferred approach. A Hospital-at-Home (HaH) program at our institution launched to provide NOM for ACD. This study evaluated the HaH program feasibility for well-selected patients.

**Methods:**

Prospective single-arm single-institution non-randomized feasibility study conducted from 10/2022 to 10/2023. Patients in the Emergency Department (ED) with ACD who met inclusion criteria (> 18 years, hemodynamically stable, caregiver at home, technology proficiency, within 10 km of service center within health network) were admitted to HaH instead of hospitalization. Participants wore devices measuring vitals, were contacted by a nurse TID, and had a virtual physician visit ≥ qd. At-home phlebotomy, IV fluids/antibiotics , priority outpatient imaging, percutaneous drainage, and PICC were available. The program ended once patients met discharge criteria or were transferred to the ward for planned surgery. Primary outcome was feasibility defined as 60% of participants *not* requiring unplanned healthcare visits 30 days post-discharge. Secondary outcomes included descriptive data, hospital bed-days saved, and unplanned healthcare visits at 60 days.

**Results:**

Of 22 patients who met inclusion criteria, 10 were enrolled. Participants were 80% male, with mean age of 56 ± 9.3 years, and mean Charlson Comorbidity Index of 1.6 ± 1.4. CT revealed abscess (3.14 ± 1.7 cm) and extraluminal gas in 90 and 40% of participants, respectively. 70% received IV antibiotics alone, 10% required percutaneous drainage, and 20% required surgery. Of the 10, 6 had resolution without further care, 2 progressed to semi-elective surgery, and 2 failed the program for ED presentation within 7 days of discharge. The HaH program had an 80% success rate. Median length of stay was 9.5 (IQR 6.3–13) days, resulting in 98 saved hospital bed-days.

**Conclusion:**

Our novel HaH program for patients with ACD demonstrated feasibility with 80% of patients successfully completing it, suggesting that HaH is a viable alternative to inpatient care for ACD in well-selected patients.

**Supplementary Information:**

The online version contains supplementary material available at 10.1007/s00464-025-12048-x.

In the United States, acute diverticulitis is the third most common inpatient gastrointestinal diagnosis with approximately 300,000 hospitalizations, resulting in 1.5 million days of inpatient care and incurs costs of over $2 billion annually [1–3]. The DIVER trial conducted in 2014 confirmed that acute uncomplicated diverticulitis could be safely managed on an outpatient basis with oral antibiotics [4]. Most current admissions on the surgical ward for acute diverticulitis at our institution (Jewish General Hospital–JGH) are composed of patients receiving intravenous antibiotics, of whom a small percentage also undergo percutaneous drainage. Overall, most days spent in hospital during these admissions are not days during which patients are receiving active procedural care.

While Hospital-at-Home (HaH) programs have successfully been described as early as the 1990s [5], they generated an unprecedented level of interest during the COVID-19 pandemic. At the JGH, this led to the creation of a Hospital-at-Home (HaH) for patients with COVID-19 seen in the Emergency Department (ED) deemed stable enough to return home with a short period of virtual monitoring. While at home, the patient wore electronic devices that regularly measured their vital signs [6]. Several times a day, a doctor and nurse contacted the patient by phone or digital visual platform to check on their progress. Alternatively, the patient could make contact on a 24/7 basis if questions or concerns arose.

In a retrospective cohort study conducted at our institution, we found that 98% of admissions for ACD between 2018 and 2022 were for patients who required intravenous antibiotics, with only 12.6% having required percutaneous drainage and 3.3% who underwent urgent surgery in the same admission [7]. Through retrospective chart review of admissions during this time period, patients who were initially managed non-operatively for ACD and met later-developed HaH admission criteria were identified as “HaH eligible.” Of 1,066 total hospital bed-days incurred by these HaH eligible ACD admissions, 827, 185, and 54 were idle, HaH, and inpatient bed-days, respectively. We defined *idle-bed days* as days spent in hospital with no diagnostic/interventional procedures, *HaH-bed days* as days where outpatient procedures available within HaH occurred, and *inpatient bed-days* as days where patients had to physically be in hospital (surgery, total parenteral nutrition). The conclusion that followed was that a HaH program for non-operative management of ACD could have potentially saved 1012 hospital bed-days during the study period.

The goal of this study was to assess the feasibility and outcomes of ACD patients managed through HaH at our institution. Our primary objective was to determine the feasibility of a HaH program for ACD patients as defined by 60% of participants not requiring unplanned healthcare visits 30 days post-discharge. Our secondary objective was to characterize the patients successfully enrolled in HaH and the care they received, describe outcomes of HaH admissions, including the number of hospital bed-days saved and assess the number of unplanned healthcare visits at 60 days post-discharge. We hypothesized that such a program could be feasible and safe in a carefully selected population suffering from acute diverticulitis.

## Methods

This was a prospective single-arm, single-institution, non-randomized feasibility study conducted from October 2022 to October 2023 approved by Review Ethics Board (REB) at the Jewish General Hospital (JGH) reported in keeping with the STROBE checklist (Supplement 1).

**Fig. 1:**
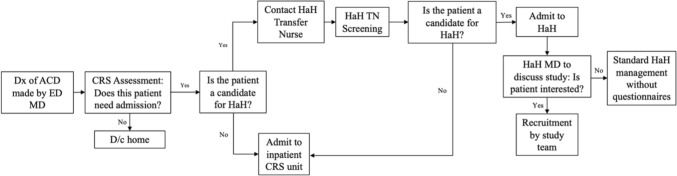
Enrollment flow of patients to HaH and feasibility study. *Dx* diagnosis, *ACD* acute complicated diverticulitis) *ED MD* Emergency Department physician, *CRS* colorectal surgery, *D/c* discharge, *HaH* Hospital-at-Home, *TN* transfer nurse, *HaH MD* Hospital-at-Home physician

Patients who received a CT imaging confirmed diagnosis of ACD after seeing an emergency physician were subsequently assessed by the Colorectal Surgery service. If deemed to meet study inclusion criteria, they were then be screened by the HaH Transfer Nurse (TN), who assessed whether the patient’s social situation was adequate for a HaH admission: social support, proficiency with technology, residing within 10 km of a local community service center within our integrated healthcare network, and living with another adult or having an available adult caregiver for the duration of the admission. After consenting to participate in the study, participants wore devices (Biobeat™ wrist and/or chest monitors, thermometers) that measured their vitals 3 times a day or more, accordingly, while at home. The HaH nursing team is composed of multiple experienced registered nurses who have received training about the particularities of caring for ACD patients. Participants were be contacted by a nurse via phone or audiovisual platform several times a day (at least three) and by the rounding physician from the Colorectal Surgery team at least once a day with the nurse present. They had the possibility of making contact with the HaH nursing team via telephone 24/7 for questions or concerns. Diagnostic and therapeutic interventions (e.g., imaging, intravenous antibiotics, percutaneous drainage, etc.) were arranged, as per standard of care, via priority-reserved same- or next-day outpatient visits. The HaH intervention ended once patients met discharge criteria or required admission to the inpatient hospital ward (Fig. 1).

### Inclusion criteria

- Adult (≥ 18 years) patients
- Image-confirmed diagnosis of ACD
  o ACD defined as acute diverticulitis of Hinchey stage Ib, II, III, and IV with findings associated with but not limited to [8] pericolic inflammation, pericolic phlegmon or abscess, distant intra-abdominal, pelvic or retroperitoneal phlegmon or abscess and finally, free air or fluid not confined to an abscess cavity.
- Admitted to HaH directly from the ED
- No indication for immediate surgery and hemodynamically stable.

### Exclusion criteria

- Inability to speak/comprehend English/French or give informed consent
- Unavailable caregiver assistance.
- Peritonitis or need for urgent surgical intervention.
- Inability to tolerate a clear liquid diet.

Feasibility of the HaH program was defined as 60% of participants *not* requiring unplanned healthcare visits within 30 days of admission to HaH. Unplanned healthcare visits are defined as visits to the Emergency Department (whether advised by a member of the treating team or not) and unplanned or urgent visits to the Colorectal Surgery clinic that are not part of scheduled patient follow-up. The current literature offers no benchmark of success for HaH for ACD, as this is a novel concept; however, described success rates for HaH for perforated appendicitis and same-day discharge colectomy vary between 57 and 70%, from which we estimated a success of 60% [9, 10]. Secondary outcomes included characterization of patients successfully enrolled in HaH and the care they received, descriptive data of HaH admissions, including the number of hospital bed-days saved, and finally, number of unplanned healthcare visits at 60 days post-discharge. All data analysis was performed using R Studio (R Core Team (2022). R: A language and environment for statistical computing. R Foundation for Statistical Computing, Vienna, Austria.) Categorical variables were reported as frequency and percentage, while continuous variables were expressed as either mean and standard deviation or median and interquartile range.

## Results

**Fig. 2:**
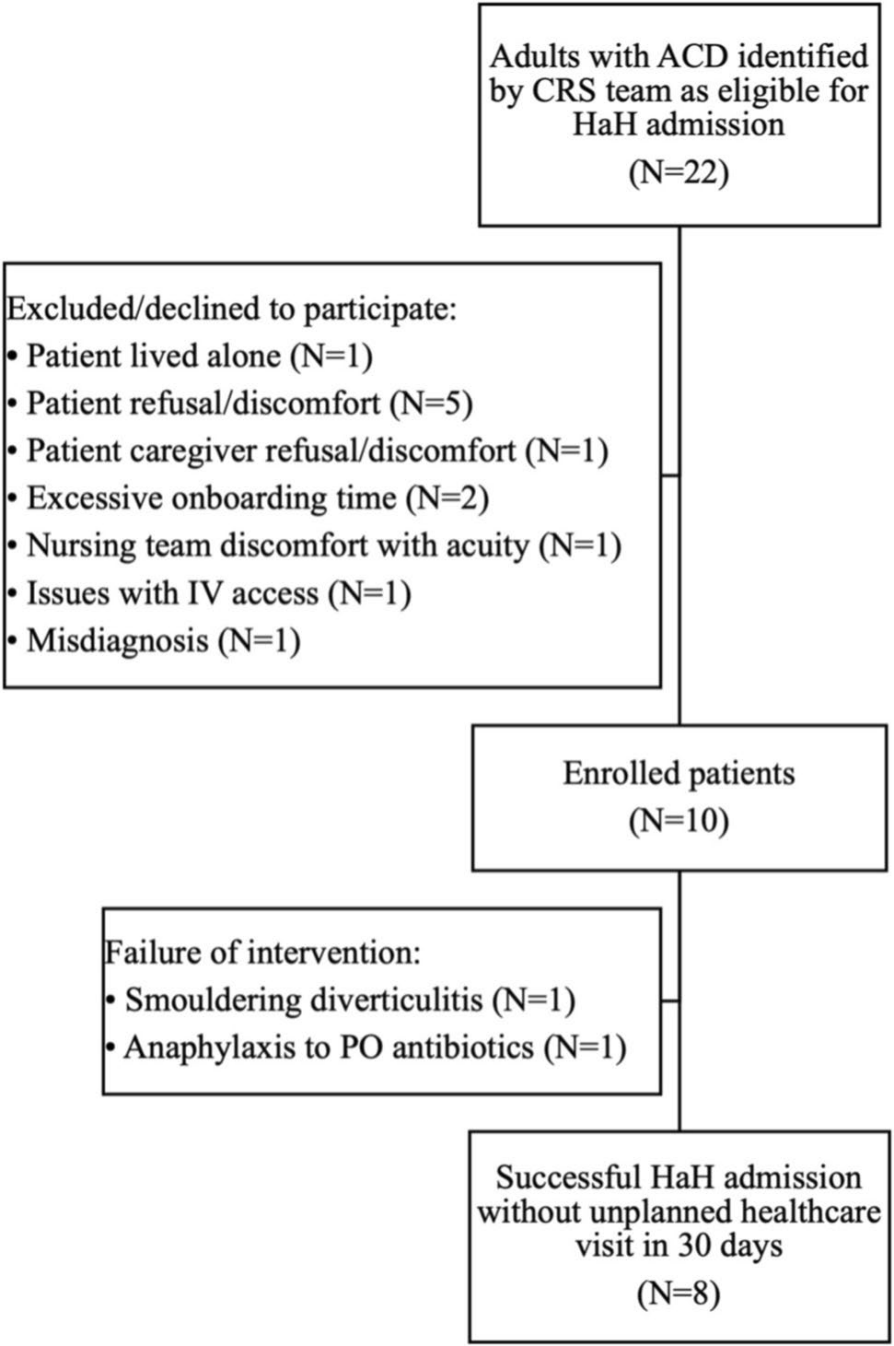
Study cohort (CONSORT flow diagram). *ACD* acute complicated diverticulitis, *CRS* colorectal surgery, *HaH* Hospital-at-Home, *IV* intravenous, *PO* per os

Of 22 patients who met inclusion criteria and were approached to enroll in the study, 12 were excluded or declined to participate for the following reasons: patient caregiver traveling during admission which would result in them living alone for period of time, patient refusal/ or discomfort with perceived level of safety, patient caregiver refusal or discomfort with perceived level of safety, excessive onboarding time to HaH, nursing team discomfort with acuity of patient, issues with IV access, and delayed identification of misdiagnosis. Thus, 10 participants were enrolled in the study **(**Fig. 2**)**, 80% of which were male, with a mean age of 56.4 ± 9.3 years, a mean body mass index (BMI) of 27.7 ± 3.5 kg/m^2^, a mean Charlson Comorbidity Index of 1.6 ± 1.4, and 30% had prior diverticulitis history (Table 1).

**Table 1:** Patient characteristics at admission (*n* = 10)

	Mean ± SD; *N* (%)
Age at admission, years	56.4 ± 9.3
Male	8 (80%)
BMI, kg/m^2^	27.7 ± 3.5
CCI	1.6 ± 1.4
Smoker	2 (20%)
Immunosuppression	0
Prior history of diverticulitis	3 (30%)
Family history of diverticulitis	3 (30%)

*BMI* body mass index, *CCI* Charlson Comorbidity Index

In this cohort, mean white blood cell count (WBC) and C-reactive protein (CRP) were 14.7 × 10^9^/L and 134.7 mg/L, respectively, on presentation. Initial CT imaging revealed predominantly Hinchey Ib disease (70%), an abscess in 90% of patients and extraluminal gas in 40% of participants. Mean abscess size was 3.14 ± 1.7 cm, and the majority of them were pericolic or intramural at 40 and 30%, respectively, with only 20% of scans showing distant abscesses. During their HaH admission, 70% of participants only required IV antibiotics, while 10% also required percutaneous abscess drainage and 20% required semi-elective surgery (Table 2).

**Table 2:** Characteristics of admissions for acute complicated diverticulitis (*N* = 10)

	*N* (%)
Hinchey (%)	
0	0
IA	1 (10%)
IB	7 (70%)
II	2 (20%)
Abscess	9 (90%)
≥ 5 cm	1 (10%)
Pericolic	4 (40%)
Intramural	3 (30%)
Pelvic/Distant	2 (20%)
Extraluminal gas	4 (40%)
Pericolic	3 (30%)
Distant	1 (10%)
Percutaneous drainage	1 (10%)
Operative management	2 (20%)

Of the 10 participants, 6 had ACD resolution without further care. Only one patient (10%) required percutaneous drainage of an intra-abdominal abscess. Two patients (20%), including the one who had undergone percutaneous drainage, were planned for semi-elective same-admission surgery and were transferred to the hospital ward after appropriate preoperative optimization within the HaH program. Unfortunately, two patients (20%) failed the program and had an unplanned healthcare visit, more specifically presented to the ED, within 7 days of discharge. The first one was for failure of disease resolution and worsening of symptoms on discharge. The second was for anaphylaxis to oral antibiotics—the first dose intake was done during the purview of the HaH admission, but their second dose which triggered the anaphylaxis was taken after discharge.

As such, the HaH program had an overall 80% success rate. The median LOS within HaH was 9.5 (IQR 6.3–13) days, and these HaH admissions resulted in 98 saved hospital bed-days.

## Discussion

In this prospective feasibility study, we found that a Hospital-at-Home (HaH) program for non-operative management of acute complicated diverticulitis (ACD) was both feasible and effective in a carefully selected patient population. Our results demonstrated an 80% success rate, exceeding our predefined feasibility threshold of 60%, with a median HaH length of stay (LOS) of 9.5 days. Importantly, the implementation of this program resulted in 98 saved in-hospital bed-days, a significant reduction in resource utilization. These findings support the notion that HaH could be a viable alternative to traditional inpatient care for selected patients with ACD, particularly in an era where hospital bed availability remains a critical issue.

The study demonstrated that HaH could be successfully implemented for ACD patients, with the majority (80%) completing their care without requiring unplanned healthcare visits. This aligns with previously reported success rates of HaH programs for other surgical conditions such as perforated appendicitis and same-day discharge colectomy, which range from 57 to 70%. Our findings suggest that, when appropriately triaged, patients with ACD can be safely managed at home with remote monitoring and virtual physician oversight.

Of the 10 patients enrolled, two required escalation of care: one for failure of disease resolution and another for anaphylaxis to antibiotics. These instances highlight the importance of careful patient selection and ongoing monitoring, as well as the need for clear escalation pathways. Notably, our study identified that 20% of participants ultimately required semi-elective surgery, suggesting that HaH may serve as a bridge to definitive intervention in select cases rather than a complete alternative to inpatient management. This was not determined a failure of the HaH program as they were scheduled for surgery and listed on the emergency list but remained home for the preoperative optimization period within the HaH virtual ward.

Traditional inpatient care for ACD involves prolonged hospital stays, primarily for administration of intravenous antibiotics and observation rather than active procedural intervention. Our study found that a substantial portion of hospital days in traditional care models are “idle-bed days,” where patients are stable but remain hospitalized. By shifting these patients to HaH, we effectively reallocated hospital resources without compromising patient safety or outcomes. The ability to conduct outpatient percutaneous drainage further supported the feasibility of HaH, as only one patient required this intervention during the study period. The potential benefits of HaH extend beyond individual patient care to broader healthcare system efficiencies. Given the increasing burden of diverticular disease on healthcare systems, HaH could serve as a strategy to optimize hospital resource utilization, particularly during periods of high demand, such as the COVID-19 pandemic. Our study’s findings suggest that even a small-scale HaH program can lead to meaningful hospital bed savings, supporting its expansion for ACD and potentially other non-operative surgical conditions.

However, challenges remain, including the need for robust home monitoring infrastructure, patient and caregiver education, and adequate staffing for remote clinical oversight. A comprehensive cost-effectiveness analysis should be conducted to ensure that the financial benefits of reducing hospital admissions are not offset by increased out-of-pocket expenses for patients, thereby shifting the economic burden rather than reducing overall healthcare costs.

Despite its promising findings, our study has limitations. The sample size was small (*n* = 10), limiting the generalizability of results. Additionally, selection bias may have influenced outcomes, as only carefully screened and consented patients were included. While this was necessary to ensure patient safety, it may not fully reflect the broader ACD patient population. Another limitation was the relatively short follow-up period, as outcomes beyond 60 days were not assessed. Future studies with larger cohorts and extended follow-up will be necessary to confirm long-term safety and efficacy, as well as evaluate patient and provider satisfaction to determine the acceptability and implementation of this model of care.

## Conclusion

Our study provides preliminary evidence that a HaH program for ACD is feasible, safe, and effective in carefully selected patients. The high success rate and substantial reduction in hospital bed-days suggest that HaH could serve as a valuable alternative to inpatient admission for select patients. Moreover, it showed that escalation of care to semi-elective surgery can be successfully and safely managed with HaH in the preoperative period. Further research is needed to refine patient selection criteria, optimize resource allocation, and evaluate long-term outcomes and cost-effectiveness. Nonetheless, these findings lay the groundwork for broader implementation of HaH models in the management of diverticular disease and other non-operative surgical conditions.

## Supplementary Information

Below is the link to the electronic supplementary material.

Supplementary file1 (DOCX 25 KB)
